# Acupuncture combined with Chinese herbal medicine on non-alcoholic fatty liver disease: A protocol for systematic review and meta-analysis

**DOI:** 10.1097/MD.0000000000028083

**Published:** 2021-12-23

**Authors:** Yuan Ma, Kewei Sun, Jianzhong Cao, Xiaowu Qin, Jiaoling Shi, Huiying Li, Jun Zhang, Tao Zhang

**Affiliations:** a Graduate School, Hunan University of Chinese Medicine, China; b Department of Liver Disease, The First Hospital of Hunan University of Chinese Medicine, China; c The School of Chinese Medicine, Hunan University of Chinese Medicine, China.

**Keywords:** acupuncture, Chinese herbal medicine, meta-analysis, non-alcoholic fatty liver disease, protocol, systematic review

## Abstract

**Background:**

Non-alcoholic fatty liver disease (NAFLD) is a global health burden. However, there are no approved drugs for NAFLD. A number of studies have shown that acupuncture combined with Chinese herbal medicine (CHM) can be beneficial for NAFLD. However, high-quality trials are still lacking. Therefore, we aimed to conduct a systematic review and meta-analysis to assess the effectiveness and safety of acupuncture combined with CHM for NAFLD.

**Methods:**

Eight electronic databases including PubMed, the Cochrane Library, Web of Science, EMBASE, China National Knowledge Infrastructure, Chinese Scientific and Technical Journals Database, and Wan-fang Database from inception to November 2021 will be searched. We will also search for Clinical Trials Registry Platforms as a supplement. Randomized controlled trials on acupuncture combined with CHM for NAFLD will be included. Literature screening, data extraction, and risk of bias assessment were independently conducted by 2 reviewers. All differences between the 2 reviewers will be discussed and resolved by a third reviewer. Revman5.3 software will be used for meta-analysis.

**Result:**

This study aimed to evaluate the effectiveness and safety of acupuncture combined with CHM for NAFLD.

**Conclusion:**

The findings of this study will provide more evidence to determine whether acupuncture combined with CHM for NAFLD is an effective and safe intervention for NAFLD.

## 1 Introduction

Non-alcoholic fatty liver disease (NAFLD), also called metabolic dysfunction-associated fatty liver disease, is the most common form of chronic liver disease and affects 25% of the global adult population.^[1,2]^ The number of NAFLD cases is increasing in parallel with the high prevalence of obesity worldwide. NAFLD covers the spectrum of liver disease, ranging from simple steatosis to non-alcoholic steatohepatitis, fibrosis, cirrhosis, and hepatocellular carcinoma, and it is predicted to be the leading cause of chronic liver disease for liver transplant in the next decade.^[3]^ Compelling evidence indicates that NAFLD is closely associated with the risk of incident type 2 diabetes, cardiovascular diseases, chronic kidney disease, and colorectal tumors.^[4]^ Active intervention for NAFLD not only prevents the development of liver disease, but also blocks the progression of metabolic syndrome.^[5]^ Insulin sensitizers, lipid-lowering drugs, antioxidants, and hepatoprotective agents are conventional pharmacological interventions for NAFLD. However, there are no approved drugs for NAFLD. Lifestyle modifications remain the first-line therapy.^[6]^ As most patients cannot achieve weight loss through lifestyle modifications, safe, economical, and effective treatments are an unmet clinical need.

Acupuncture treatment is a non-pharmacological therapy that uses needles to stimulate acupoints with different manipulation techniques to treat diseases according to the theory of Chinese medicine. Although a number of clinical studies have indicated that acupuncture may effectively treat NAFLD by promoting hepatic lipid metabolism, reducing insulin resistance, and improving liver function,^[7,8]^ the effectiveness of acupuncture in NAFLD remains controversial because of the lack of high-quality randomized controlled trials (RCTs). Chinese herbal medicine (CHM), mainly from plant parts, has attracted increasing attention for the treatment of NAFLD in recent years.^[9–11]^ Pre-clinical and clinical research has shown that CHM has good effects in treating NAFLD.^[12–15]^ However, CHM also has a risk of adverse effects and toxicity, similar to other drugs.

Acupuncture combined with CHM is widely used to treat NAFLD in China, and combination therapy is considered to provide better therapeutic effect and less toxicity in the treatment of NAFLD compared with monotherapy in most patients. However, sufficient evidence is still lacking. Therefore, we conducted a systematic analysis and meta-analysis to evaluate the effectiveness and safety of acupuncture combined with CHM for NAFLD.

## 2 Methods

### 2.1 Study registration

This protocol was registered on open science framework (DOI: 10.17605/OSF.IO/8GYVC). It was completed in accordance with the Preferred Reporting Items for Systematic Review and Meta-Analysis Protocols (PRISMA-P).^[16]^

### 2.2 Inclusion and exclusion criteria for this study

#### 2.2.1 Types of studies

All RCTs on acupuncture combined with CHM for NAFLD published in Chinese and English will be included.

#### 2.2.2 Types of participants

Adult patients (18 years old and above) with established NAFLD based on histologic or imaging evidence regardless of sex, race, nationality, educational background, and medical units will be included.

#### 2.2.3 Types of intervention

The intervention group was treated with acupuncture combined with CHM.

#### 2.2.4 Types of comparison

The control group was given acupuncture or CHM alone.

#### 2.2.5 Outcomes

(1) Primary outcomes: ① total effective rate, ② liver function (alanine transaminase ALT, aspartate transaminase AST, gamma-glutamyl transferase GGT), ③ serum lipid (total cholesterol TC, triglyceride TG), and ④ liver B-ultrasound.

The efficacy of the patients will be evaluated according to the guidelines for the prevention and treatment of NAFLD (2018, China).^[4]^ Clinical cure: liver function and blood lipid indexes returned to normal; remarkable effective: liver function index decreased by more than 50%, TC decreased by ≥20%, or TG decreased by ≥40%; effective: liver function index decreased by 30% to 50%, TC decreased by 10% to 20%, or TG decreased by 20% to 40%; invalid: liver function index did not improve and blood lipid levels were not reduced.

Total effective rate = (clinical cure + remarkable effective + effective) cases/total cases × 100%.

(2) Secondary outcomes: ① Homeostasis model assessment for insulin resistance, ② body mass index, and ③ adverse events.

#### 2.2.6 Exclusion criteria for this study

(1) Reviews, comments, case reports, animal or cell line experiments.
(2) Studies with incomplete data or duplicate publications.
(3) Fatty liver disease caused by autoimmune liver disease, viral hepatitis, alcoholic liver disease, and other liver diseases.
(4) NAFLD combined with serious cardiovascular and cerebrovascular diseases, and malignant tumors.

### 2.3 Data search strategy

Electronic databases including PubMed, the Cochrane Library, Web of Science, EMBASE, China National Knowledge Infrastructure, Chinese Scientific and Technical Journals Database, Wan-fang Database, and Chinese Biomedical Literature Database, will be searched from inception to November 2021. In addition, we will search for other sources from the PROSPERO, Clinical Trials.gov, Chinese Clinical Trial Registry, the US National Institutes of Health register, and the World Health Organization International Clinical Trials Registry Platform.

“Non-alcoholic fatty liver disease,” “acupuncture,” “Chinese herbal medicine,” “randomized controlled trial” as the keywords will be searched. The retrieval strategy in PubMed as an example is presented in Table 1 using Medical Subject Headings (MeSH) and free text words. Similar search strategies will be applied to other electronic databases.

**Table 1:** Retrieval strategy of PubMed.

Number	Search terms
#1	“Non-alcoholic fatty liver disease”[Mesh] OR “NAFLD”[Ti/Ab] OR “fatty liver, Nonalcoholic”[Ti/Ab] OR “liver, Nonalcoholic Fatty”[Ti/Ab] OR “Nonalcoholic Steatohepatitis”[Ti/Ab] OR “Nonalcoholic Steatohepatitis”[Ti/Ab] OR “Steatohepatitis, Nonalcoholic ”[Ti/Ab]
#2	“acupuncture”[Mesh] OR “acupuncture therapy”[Ti/Ab] OR “electroacupuncture”[Ti/Ab] OR “needling therapy”[Ti/Ab] OR “acupoint therapy”[Ti/Ab] OR “abdominal acupuncture”[Ti/Ab] OR “ear acupuncture”[Ti/Ab] OR “warming acupuncture”[Ti/Ab] OR “skin acupuncture”[Ti/Ab] OR “wrist-ankle acupuncture”[Ti/Ab]
#3	“Chinese herbal medicine”[Ti/Ab] OR “herbal medicine”[Ti/Ab] OR “traditional Chinese medicine”[Ti/Ab] OR “TCM”[Ti/Ab] OR “herbals”[Ti/Ab] OR “CHM”[Ti/Ab] OR “Chinese Medicine”[Ti/Ab]
#4	#2 AND #3
#5	“randomized controlled trial”[Mesh] OR “controlled clinical trial”[Mesh] OR “randomly”[Ti/Ab] OR “randomized”[Ti/Ab] OR “RCT”[Ti/Ab] OR “clinical trial”[Ti/Ab]
#6	#1 AND #4 AND #5

### 2.4 Study selection and data extraction

**Figure 1:**
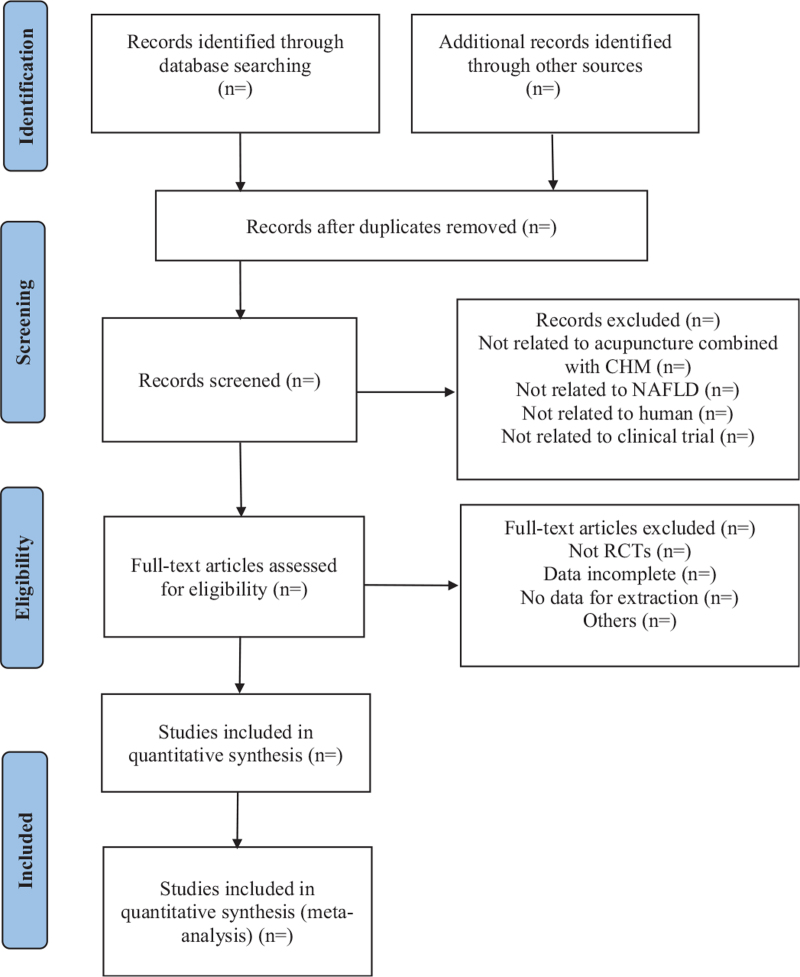
Flow chart of literature selection process. CHM = Chinese herbal medicine, NAFLD = non-alcoholic fatty liver disease, RCTs = randomized controlled trials.

According to the retrieval strategy, all the studies searched from the database will be input into Endnote X9.3 (The Thomson Corporation Corp, Stanford, CT), where deduplication will be conducted. Two reviewers (YM and XQ) will independently choose related studies after reading the titles, abstracts, and keywords. The remaining documents will be screened by reading the full text. All differences between the 2 reviewers will be discussed and resolved by a third reviewer (KS). We will contact the author if the required information is missing. The detailed selection process is shown in Figure 1.

Two reviewers (HL and JS) will independently extract data using a pre-determined data extraction table. The contents include general data (title, author, publication date, and literature source), participants (number of cases, age, sex, and disease course), interventions (intervention measures, intervention time and frequency, follow-up duration), and outcomes (total effective rate, ALT, AST, GGT, TC, TG, liver B-ultrasound, homeostasis model assessment for insulin resistance, body mass index, and adverse events). The collected data were managed using Microsoft Excel 2016 (Microsoft Corporation, Redmond, WA).

### 2.5 Assessment of the risk of bias

Based on the Cochrane Handbook for Systematic Reviews of Interventions V5.1.0, 2 authors (YM and XQ) independently evaluated the risk of bias in eligible studies. The items included random sequence generation, allocation concealment, blinding methods, incomplete data, selective outcome reporting, and other biases. Each subject will be categorized into 3 levels “low bias,” “unclear,” “high bias.” When disagreements arise between the 2 researchers, they should discuss and hold counsel with a third reviewer (KS).

### 2.6 Statistical analysis

Revman5.3 (The Cochrane Collaboration, Copenhagen, 2014) software will be used for meta-analysis. Relative risk with 95% confidence interval will be used to measure the curative effect for dichotomous variables, and the mean difference or standardized mean difference with 95% confidence interval will be used for continuous data. We will use the chi-square test and *I*^*2*^ statistic to evaluate the heterogeneity of the included studies. If there is no statistical heterogeneity (*P* *>* .1 or *I*^*2*^ *<* 50%), a fixed-effect model will be applied. Otherwise, a random-effects model will be used for meta-analysis, excluding obvious clinical heterogeneity. We will use a subgroup analysis or sensitivity analysis for clinical heterogeneity. Narrative synthesis will be performed if meta-analysis is not inappropriate.

#### 2.6.1 Subgroup analysis

Subgroup analysis will be carried out according to the information of patients (age, sex, disease course), intervention measures, intervention time, and frequency if enough studies have been included.

#### 2.6.2 Sensitivity analysis

The objective of the sensitivity analysis is to evaluate the stability and robustness of the primary results. Sensitivity analysis can be carried out in several ways, such as reducing the literature one by one to see if the final conclusion has changed, changing a statistical method, and deleting low-quality studies.

#### 2.6.3 Reporting bias

When we have adequate samples, a funnel plot will be applied to evaluate reporting bias with a symmetric funnel plot representing a low risk of bias and an asymmetric funnel plot representing a high risk of bias.

### 2.7 Grading the quality of evidence

The Grading of Recommendation, Assessment, Development and Evaluation (GRADE) system^[17]^ is used to appraise the quality of evidence from the studies obtained. Their levels are divided into high, moderate, low, and very low levels.

### 2.8 Dissemination and ethics

The data of this review will be derived from published studies, and ethical approval is not required. The results of this review will be published after completion.

## 3 Discussion

NAFLD has become a global health burden. However, there are no approved pharmacological therapies for this condition. Many patients seek acupuncture and CHM treatment in China, and a number of RCTs have shown that acupuncture combined with CHM can be beneficial for NAFLD.^[18,19]^ Given that there is no meta-analysis to evaluate the existing evidence, we conducted this systematic analysis and meta-analysis to evaluate the effectiveness and safety of acupuncture combined with CHM on NAFLD in order to provide more evidence for its clinical application.

Although we have developed a detailed protocol for this systematic review and meta-analysis, there are still some limitations to this review. First, we only included literature in English and Chinese, and this may cause a risk of bias. Second, acupuncture treatment includes different acupoints and acupuncture methods, and CHM also contains different dosage forms. They may cause clinical heterogeneity and influence the results of this study. Subgroup analysis, sensitivity analysis, and funnel plot will be performed if the literature is sufficient to reduce clinical heterogeneity and obtain better evidence.

## Author contributions

**Conceptualization:** Yuan Ma, Kewei Sun.

**Data curation:** Yuan Ma, Xiaowu Qin.

**Funding acquisition:** Kewei Sun, Tao Zhang.

**Investigation:** Jiaoling Shi, Jun Zhang.

**Methodology:** Jiaoling Shi, Huiying Li.

**Supervision:** Jiaoling Shi, Jun Zhang.

**Validation:** Huiying Li, Tao Zhang.

**Writing – original draft:** Yuan Ma.

**Writing – review & editing:** Yuan Ma, Kewei Sun, Jianzhong Cao, Tao Zhang.
